# Anti-inflammatory CAR-microglia targeting Aβ for Alzheimer’s disease therapy

**DOI:** 10.3389/fimmu.2026.1820099

**Published:** 2026-07-14

**Authors:** Xizhong Ding, Xukai Hu, Wanqiang Xue, Yingrui An, Wei Cheng, Shaolong Zhang, Anhua Lei, Jin Zhang

**Affiliations:** 1 Liangzhu Laboratory & Center for Stem Cell and Regenerative Medicine, Department of Basic Medical Sciences & Bone Marrow Transplantation Center of the First Affiliated Hospital, Zhejiang University School of Medicine, Hangzhou, China; 2 Institute of Health and Medicine, Hefei Comprehensive National Science Center, Hefei, China; 3 Center of Gene/Cell Engineering and Genome Medicine of Zhejiang Province, Hangzhou, China; 4 Transvascular Implant Instrument Research Institute, the Second Affiliated Hospital, Zhejiang University School of Medicine, Hangzhou, China

**Keywords:** Alzheimer’s disease, amyloid-β, chimeric antigen receptor, iPSC, microglia, neuroinflammation

## Abstract

Alzheimer’s disease (AD) is characterized by the accumulation of amyloid-β (Aβ) plaques and chronic neuroinflammation, which together drive progressive neuronal loss and cognitive decline. In recent years, monoclonal antibodies targeting Aβ have demonstrated encouraging clinical benefits in Alzheimer’s disease (AD). However, their therapeutic efficacy remains limited by insufficient and unsustained clearance of Aβ, as well as treatment-associated neuroinflammatory responses. These limitations highlight the need for alternative strategies that can achieve efficient Aβ elimination while maintaining immune homeostasis. To overcome these challenges, we developed a novel anti-inflammatory CAR-Microglia (CAR-Mic) incorporating a construct based on the TAM receptor family (TYRO3, AXL, and MERTK), which are key regulators of efferocytosis and anti-inflammatory responses. The resulting Aβ-targeted CAR-Mics showed enhanced Aβ engulfment and reduced proinflammatory cytokines release. Among the constructs tested, AXL-CAR demonstrated the most favorable overall performance and was therefore selected for the generation of human induced pluripotent stem cell (iPSC)-derived CAR microglia-like cells (CAR-iMGLs). In an AD mouse model, AXL-CAR-iMGLs exhibited enhanced Aβ clearance without evidence of severe adverse effects. Collectively, these findings establish TAM receptor-based CAR-iMGLs as a promising cell therapy model for AD and potentially other neurodegenerative disorders characterized by chronic neuroinflammation and defective pathological protein clearance.

## Introduction

Alzheimer’s disease (AD) is characterized by the progressive accumulation of amyloid-β (Aβ) plaques and neurofibrillary tangles, accompanied by chronic neuroinflammation, neuronal dysfunction, and neurodegeneration (1, 2). In recent years, monoclonal antibodies targeting Aβ have achieved promising clinical outcome (3–5), however, these antibodies predominantly engage Fc receptors on microglia, often leading to excessive immune activation and neuroinflammation, and inducing synapse loss and cognitive deficits (6–8). Moreover, microglia in patients with AD often exhibit impaired capacity to phagocytose and degrade Aβ, due to genetic risk variants and prolonged exposure to inflammatory stimuli, which may limit the sustained effectiveness of antibody-based therapies (9–11).

Adoptive transfer of microglia or myeloid cells has recently emerged as an alternative strategy to enhance Aβ or Tau clearance in AD (9, 12, 13). Several studies have shown that transplantation of exogenous microglia or monocyte-derived macrophages into AD mouse models can reduce amyloid burden (9, 13, 14). However, due to the plasticity of myeloid cells, transplanted cells are readily reshaped by the inflammatory microenvironment of the AD brain, often acquiring a pro-inflammatory phenotype that undermines long-term therapeutic efficacy. Engineering microglia with chimeric antigen receptors (CARs) offers a rational approach to overcome these limitations by conferring defined antigen specificity and programmable intracellular signaling (15). However, the optimal CAR architecture that simultaneously supports efficient Aβ phagocytosis and anti-inflammatory ability within the central nervous system remains to be established.

The TAM (TYRO3, AXL, and MERTK) family of receptor tyrosine kinases has been identified as a central regulator of efferocytosis and immune homeostasis (16–18). TAM receptors share a conserved architecture consisting of two extracellular immunoglobulin-like domains, two fibronectin type III repeats, a single transmembrane helix, and an intracellular kinase domain. Their ligands, Gas6 and Protein S, bind phosphatidylserine on apoptotic cells and bridge TAM dimerization and activation. Downstream signaling induces STAT1-dependent expression of suppressor of cytokine signaling (SOCS) proteins, broadly attenuating cytokine and Toll-like receptor pathways (18). Through this mechanism, TAM signaling tightly couples phagocytic clearance with negative feedback control of inflammation. In AD brains, Axl and Mertk are highly expressed in microglia around Aβ plaques, and their activation by Gas6/Protein S is essential for plaque detection and engulfment (17, 19). A previously reported chimeric Gas6 fusion protein targeting amyloid-β (αAβ–Gas6) significantly reduces Aβ burden through TAM receptor–dependent phagocytosis without eliciting inflammatory responses (20). These studies implicate TAM signaling as a critical regulator of both Aβ clearance and the restraint of neuroinflammation.

Here, we report the rational design and functional screening of an anti-inflammatory CAR platform for microglia, aimed at enhancing amyloid-β (Aβ) clearance while constraining neuroinflammatory activation. By systematically engineering Aβ-specific CARs incorporating intracellular signaling domains derived from the TAM receptor family, we identified an AXL-based CAR structure that possessed efficient Aβ clearance and anti-inflammatory capacity. Using both immortalized myeloid cell lines and human iPSC-derived microglia-like cells, we demonstrated that AXL-CAR-Mic exhibited enhanced Aβ uptake and lysosomal degradation of Aβ, accompanied by attenuation of pro-inflammatory cytokines in acute, primed, and Aβ-induced inflammatory conditions. Importantly, adoptive transfer of human iPSC-derived AXL-CAR microglia-like cells into AD mouse brains resulted in a significant reduction of Aβ plaque burden *in vivo*. Collectively, our findings establish a CAR-microglia strategy that integrates antigen-specific Aβ clearance and anti-inflammatory functions, providing a promising therapeutic strategy for Alzheimer’s disease.

## Materials and methods

### Cell culture

Human iPSCs used in this study were generated and characterized in our previous works (21, 22). iPSCs were maintained on Matrigel-coated plates (Corning, 354277) in mTeSR™-1 medium (STEMCELL Technologies, 85851). HEK293T, RAW264.7, HMC3, and BV2 cells were cultured in high-glucose DMEM (Gibco, 11965-065) supplemented with 10% fetal bovine serum (FBS; Gibco, 10099-141) and 1× penicillin–streptomycin (Gibco, 15140-122).

### Animals

All animal experiments were conducted in accordance with protocols approved by the Institutional Animal Care and Use Committee (IACUC) of the Hefei Comprehensive National Science Center Institute of Health and Medicine. B6SJL-Tg(APPSwFlLon, PSEN1*M146L*L286V)6799Vas/Mmjax (5xFAD) mice were purchased from the Jackson Laboratory. B6/JGpt-Tg(Thy-APP/Thy-PSEN1)5/Gpt (FAD4T) mice were purchased from Gempharmatech Co., Ltd (NanJing, China). C57BL/6J mice were purchased from SLAC animal (Shanghai, China). All mice were housed under specific pathogen–free (SPF) conditions.

At predetermined time points, mice were deeply anesthetized with pentobarbital sodium (200 mg/kg, intraperitoneal injection) or avertin (200 mg/kg, intraperitoneal injection), and adequate anesthesia was confirmed by loss of the righting reflex and absence of response to toe pinch. Mice were then euthanized by transcardial perfusion, followed by cervical dislocation to ensure death. Brain tissues were subsequently collected for histological and immunofluorescence analyses.

### Constructs

Two versions of chimeric antigen receptor (CAR) constructs were designed. In the V1 CAR, an aducanumab-derived scFv was fused to a CD8 hinge and transmembrane domain, followed by an intracellular signaling domain derived from the TAM receptor family. In the V2 CAR, the aducanumab-derived scFv was followed by a fibronectin type III (FNIII) domain, a transmembrane region, and a TAM receptor–based intracellular domain. All CARs were synthesized by Tsingke Biotechnology and cloned into the lentivirus vector Lenti-EF1A-T2A-EGFP-PGK-Puro vector.

To generate hCSF1-overexpressing vector, a DNA fragment encoding human CSF1 (hCSF1) fused to a G4S linker and a C-terminal His10 tag (hCSF1-G4S-His10) was synthesized by Tsingke Biotechnology. The synthesized fragment was cloned into a lentiviral expression vector under the control of the EF1α promoter, yielding the construct Lenti-EF1α-hCSF1-G4S-His10-PGK-Blast.

### Antibodies

Information for antibodies used is listed below: Rabbit anti-IBA1(1:200, ABclonal, A19776), rabbit anti-P2RY12(1:125, Sigma, HPA014518), mouse anti-β-Amyloid(clone 6e10, 1:1000, Biolegend, 803014), rabbit anti-LAMP1(1:50, ABclonal, A16894), rabbit anti- Phospho-Stat1 (Tyr701) (1:500, CST, 9167S), Alexa Fluor^®^ 647 anti-STAT1 Phospho (Ser727) (1:100, Biolegend, 686401), FITC anti-human CD43(1:100, Biolegend, 315203), goat anti-Mouse IgG H&L (AF647, 1:1000, Abcam, ab150115), goat anti-rabbit IgG H&L (AF555, 1:500, Abcam, ab150078), Rabbit anti-NeuN (1:200, ABclonal, A19086), Rabbit anti-Synaptophysin (1:200, ABclonal, A27659), PE/Cyanine7 anti-mouse/human CD11b (clone M1/70, 1:100, Biolegend, 101216), Brilliant Violet 711 anti-human CD45 (clone HI30, 1:100, Biolegend, 304049), Brilliant Violet 605 anti-mouse CD45 (clone 30-F11, 1:100, Biolegend, 103140).

### Lentivirus production

Lentiviral vectors were produced using a standard third-generation packaging system. Briefly, HEK293T cells were co-transfected with the lentiviral transfer plasmid encoding the indicated CAR constructs or hCSF1-overexpressing vector, together with the packaging plasmids psPAX2 and pMD2.G, using polyethyleneimine (PEI). Viral supernatants containing lentiviral particles were collected at 48 and 72 h post-transfection, clarified by low-speed centrifugation and filtration through a 0.45 μm filter, and either used directly or concentrated using PEG8000. Viral titers were determined by transduction of HEK293T cells followed by flow cytometric analysis of reporter expression.

### Generation of hCSF1-expressing CAR-iMGLs from human iPSC

For hCSF1 transduction, human iPSCs were passaged at approximately 80% confluency at a 1:10 split ratio. The following day, cells were transduced with Lenti-EF1α-hCSF1-G4S-His10-PGK-Blast lentivirus in mTeSR1 medium containing 5 μg/mL polybrene. After 12 h, the medium was replaced with fresh mTeSR1. At 48 h post-transduction, cells were selected with 10 μg/mL Blasticidin S (Gibco) for 7 days to generate stable hCSF1-overexpressing iPSC lines.

For CAR transduction, hCSF1-overexpressing human iPSCs were passaged at approximately 80% confluency at a 1:10 ratio. The following day, human iPSCs were transduced with lentiviral vectors encoding the CAR construct at an MOI of 10 in mTeSR-1 medium supplemented with polybrene (5 μg/mL). After 12 hours transduction, the medium was replaced with fresh mTeSR-1 medium. At 48 h post-transduction, EGFP fluorescence was examined under a fluorescence microscope to confirm successful lentiviral transduction. When the cells reached approximately 80% confluency, EGFP-positive cells were enriched by flow cytometric sorting. Prior to sorting, the transduction efficiency was approximately 30%–50%, as determined by EGFP positivity. After sorting, the EGFP positivity was approximately 90%. CAR-expressing iPSCs were differentiated into microglia-like cells according to a previously published protocol (22). On day 0, iPSCs were dissociated by TrypLE and 8, 000 cells were seeded into 96-well round-bottom plates in APEL2 medium (05271, STEMCELL Technologies) supplemented with 100 ng/mL human Stem Cell Factor (SCF), 50 ng/mL human Vascular Endothelial Growth Factor (VEGF), 10 ng/mL recombinant human Bone Morphogenetic Protein 4 (BMP-4), 5 ng/mL human FGF-basic (FGFb), and 10 μM Rho kinase inhibitor (ROCK inhibitor, Y27632, Sigma). Medium were refreshed every 3 days. On day 7, cells were cultured into APEL2 medium containing 10ng/mL human FGFb, 50ng/mL VEGF, 50ng/mL human SCF, 10ng/mL recombinant human Insulin-like Growth Factor-1 (IGF1), 20ng/mL IL-3, 50ng/mL recombinant human M-CSF, and 50ng/mL recombinant human GM-CSF. On day 10, embryoid bodies (EB) were transferred into Matrigel-coated 6-well plates, and cultured in the X-VIVO15 medium, containing the same cytokine cocktail. Floating hematopoietic progenitors were collected and further differentiated in microglia differentiation medium (DMEM/F12 medium containing 2% v/v ITSG-X, 5 μg/mL insulin, 2% B27, 0.5% N2, 1% glutamax, 1% non-essential amino acids, 100 ng/mL IL-34, 50 ng/mL TGFb1, and 25 ng/mL M-CSF), as described by Abud (23) et al. Collected cells were cultured in the microglia differentiation medium for 7 days for transplantation or 28 days for *in vitro* assays, with medium changes every 3 days.

### Generation of Aβ oligomer and pHrodo conjugation

Human β-amyloid 1–42 (1 mg; MCE) was dissolved in 400 μL ice-cold HFIP, vortexed, and incubated at room temperature for 2 h. The solution was aliquoted (500 μg per tube), dried using a vacuum centrifuge, and stored at −80 °C. For oligomerization, 500 μg Aβ was resuspended in 20 μL DMSO, then 380 μL PBS was added, incubating at 4 °C for 24 h. Then 1 μL pHrodo red(Invitrogen, P36011) was added and incubated for 3 h room temperature in the dark. The conjugated oAβ–pHrodo was washed twice using a 30 kDa centrifugal filter (Amicon, UFC5030) and resuspended in 100uL 5% DMSO in PBS. The resulting preparation was characterized by western blot analysis and confirmed by 6E10 antibody immunostaining (**Supplementary Figure 5A**).

### *In vitro* oAβ-pHrodo engulfment experiment

Control or CAR-expressed cells were incubated with 5 μg/mL oAβ-pHrodo for 16 h, and analyzed by confocal microscopy or flow cytometry.

For live-cell imaging, cells were incubated with 5 μg/mL oAβ-pHrodo, and monitored by Incucyte. Images were acquired every 0.5 h for 24 h using a 20x objective lens, and 4 fields of view were captured per well. For each condition, experiments were performed with 3 biological replicates and 4 technical replicates. Error bars represent mean ± s.e.m. derived from independent biological replicates. Relative oAβ-pHrodo engulfment was calculated as the ratio of pHrodo and EGFP double-positive area (green + red fluorescence) to EGFP-positive cell area (green fluorescence), normalized to the 0 h time point.

### Cytokine measurement

Cells were starved with serum-free medium for 24 h and then stimulated under different inflammatory conditions. Acute inflammation was induced by treatment with LPS (100 ng/mL) and oAβ (5 μM) for 6 h. For primed inflammation, cells were pretreated with LPS for 2 h followed by oAβ for 4 h. Chronic inflammation was induced by oAβ treatment for 16 h. Total RNA was extracted for qPCR analysis.

### *In vitro* Aβ degradation assay

EGFP or AXL-CAR-iMGLs (5 × 10^5^) were incubated with 5 μM oAβ for 5 days. Cells and supernatants were collected and analyzed by SDS–PAGE and western blotting using anti–β-amyloid (6E10). Band intensities were quantified using Image Lab software.

### Mouse microglia depletion

Microglia were depleted by feeding mice with PLX5622-formulated AIN-76A diet (1,200 ppm) for 14 days, as previously described (24).

### Intracranial injection

Mice were anesthetized under continuous isoflurane and bilaterally injected with 2 μL of DPBS, EGFP-iMGLs or AXL-CAR-iMGLs at 50, 000 cells/μL into the hippocampus (anteroposterior, 2.06 mm; mediolateral, ± 1.75 mm; dorsoventral, 1.75 mm, relative to bregma). Injections were performed at a rate of 0.5 μL per 30 s, followed by a 4 min dwell time before needle withdrawal. The cell viability was assessed before transplantation using trypan blue, the final iMGLs preparation used for animal injection exhibited a viability of over 95%.

### Immunofluorescence

For immunofluorescence staining of cultured cells, cells were seeded onto 0.1% gelatin-coated glass coverslips and allowed to adhere overnight. Cells were fixed with 4% paraformaldehyde for 20 min at room temperature, washed three times with 1xPBS, permeabilized with 0.25% Triton X-100 in 1xPBS for 1 h at room temperature, and blocked for 30 min in blocking buffer (0.2% BSA, 0.25% Triton X-100, and 5% donkey serum in 1xPBS). Cells were then incubated with primary antibodies for 1 h at 4 °C, washed three times with PBST (0.1% Tween-20 in 1xPBS), and incubated with appropriate secondary antibodies for 1 h at room temperature in the dark. After washing, coverslips were mounted using antifade mounting medium containing DAPI (Beyotime).

For immunofluorescence staining of brain sections, mice were anaesthetized with pentobarbital sodium or avertin and transcardially perfused with 1xPBS followed by 4% paraformaldehyde. Brains were post-fixed overnight in 4% paraformaldehyde and cryoprotected in 30% sucrose in PBS for 48 h, embedded in OCT, and sectioned coronally at 30 μm using a cryostat (Leica). Sections were permeabilized with 0.25% Triton X-100 in PBS for 1 h at room temperature and incubated in blocking solution (0.2% BSA, 0.25% Triton X-100, 5% donkey serum in 1xPBS) for 1 h. Sections were then incubated with primary antibodies overnight at 4 °C, washed three times with 1xPBST, and incubated with secondary antibodies for 1 h at room temperature in the dark. After washing, sections were mounted with antifade mounting medium containing DAPI (Beyotime).

### Aβ quantification

For *in vivo* Aβ quantification, three coronal brain sections spanning the injection site (approximately −1.8 to −2.2 mm from bregma) were analyzed for each animal. The entire hippocampal region was manually delineated as the region of interest (ROI) in both hemispheres. Aβ burden was quantified as the percentage of Aβ-positive area and Aβ-plague number within the hippocampal ROI using Fiji (ImageJ). The same thresholding parameters were applied to all images within each experiment. Measurements from the left and right hippocampi were averaged to obtain a single value for each animal.

### Three-dimensional reconstruction

Z-stack confocal images were acquired using LSM900 (Zeiss) or FV3000 (Olympus) at 1-μm intervals. 3D reconstructions were generated using Imaris software. Surface rendering and volume visualization were generated using the corresponding fluorescence channels with identical display parameters applied across samples. Representative three-dimensional reconstructions were used to visualize the spatial colocalization between transplanted iMGLs and Aβ plaques.

### Flow cytometry

Flow cytometry was performed on DxFLEX (Beckman) or LSRFortessa (BD Biosciences). Data were analyzed by FlowJo(V10).

For *in vitro* cultured samples, approximately 2 × 10^5^ cells were collected and stained with the indicated antibodies for 20 min at 4 °C. After passing through a 70-μm cell strainer, the cells were analyzed by flow cytometry.

For ex vivo brain samples, mouse hippocampi were isolated on ice and dissociated in HBSS containing 400 U/mL Collagenase IV and 30 U/mL DNase I at 37 °C for 20 min with gentle agitation. The resulting cell suspension was filtered through a 70-μm cell strainer and centrifuged at 500 × g for 5 min at 4 °C. Myelin debris was removed by centrifugation in 30% Percoll at 800 × g for 20 min at 4 °C without brake. The recovered cells were washed twice with FACS buffer and stained with fluorophore-conjugated antibodies against the indicated surface markers. Prior to acquisition, DAPI was added to exclude dead cells. Total microglia were defined as CD11b-positive cells expressing intermediate levels of mouse CD45 or negative for mouse CD45, encompassing both endogenous mouse microglia and engrafted human microglia. Engrafted iMGLs were subsequently identified within the total microglia population based on co-expression of human CD45 and CD11b.

### Statistical analysis

Statistical analyses were performed using GraphPad Prism 8.4.3. The specific statistical tests applied, sample sizes (n), and measures of variability are indicated in the corresponding figure legends. Data are presented as mean ± S.E.M. Statistical significance was assessed using two-tailed Student’s *t* test, one-way ANOVA, or two-way ANOVA, as appropriate. All differences were considered significantly different when p < 0.05.

## Results

### Generation and screen of the Aβ-targeting anti-inflammatory CAR constructs

**Figure 1:**
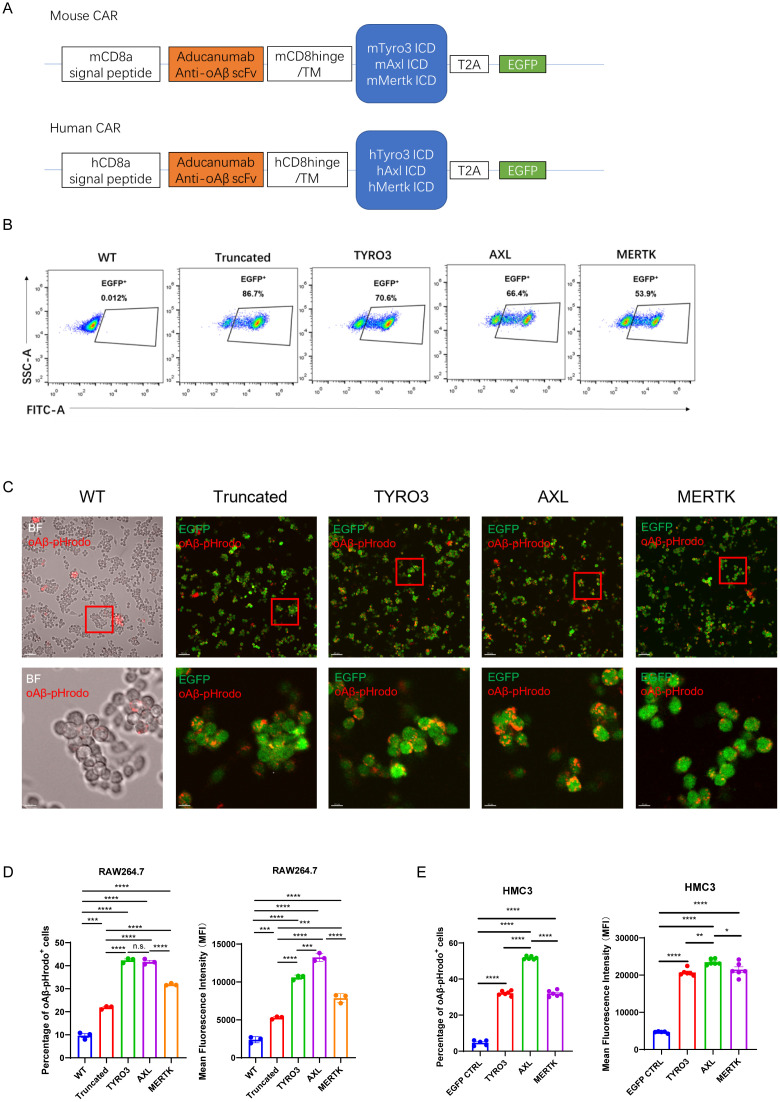
Anti-oAβ CAR constructs promoted macrophage and microglia engulfment of Aβ-oligomer *in vitro*. **(A)** Schematic representation of mouse- or human-derived oAβ-targeting CAR constructs. Each CAR comprises a CD8α signal peptide, an Aducanumab-derived scFv, a CD8 hinge and transmembrane domain, and an intracellular signaling domain from a member of the TAM receptor family. EGFP was included downstream as a reporter. **(B)** Flow cytometry analysis of CAR expression in Raw264.7 cells through the detection of EGFP reporter protein. **(C)** Representative confocal images of WT, Truncated-CAR, Tyro3-CAR, AXL-CAR and MERTK-CAR Raw264, 7 cells after incubation with oAβ-pHrodo for 16h. Scale bar 50 μm in the original images and 10 μm in the enlarged images. **(D)** Flow cytometry quantification of oAβ-pHrodo engulfment in WT, Truncated-CAR, Tyro3-CAR, AXL-CAR and MERTK-CAR Raw264, 7 cells after incubation with oAβ-pHrodo for 16h (n=3 for all conditions). **(E)** Flow cytometry quantification of oAβ-pHrodo engulfment of EGFP-CTRL, Tyro3-CAR, AXL-CAR and MERTK-CAR HMC3 cells after incubation with oAβ-pHrodo for 16h (n=5, 6, 6, 6). All data are presented as mean ± s.e.m, and significance was calculated by one-way ANOVA followed by Tukey’s multiple comparisons test (n.s., not significant; *P < 0.05; **P < 0.01; ***P < 0.001; ****P < 0.0001).

To combine antigen targeting with TAM signaling, we designed chimeric antigen receptors in which an Aβ-specific single-chain antibody fragment (scFv) was fused to the intracellular domain of a TAM receptor. We first linked the Aβ-targeting scFv domain with the intracellular domain of TAM receptors through a CD8 hinge and a CD8 transmembrane domain following the conventional CAR design strategy, and using EGFP as a reporter protein (**Figure 1A**). We also generated a construct of a truncated CAR without the intracellular domain, and a construct containing only EGFP. Different control groups were used depending on the specific experimental question. WT cells were used to define baseline phagocytic and inflammatory responses, EGFP-transduced cells served as vector controls, and truncated CAR constructs lacking intracellular signaling domains were used to dissect the contribution of TAM-mediated signaling. CAR constructs were successfully transduced into mouse monocyte/macrophage cell line Raw264.7 (**Figure 1B**).

We prepared oligomeric amyloid-β (oAβ) according to established protocols (20). The oAβ was subsequently conjugated to the pH-sensitive fluorophore pHrodo, which selectively fluoresces within acidic compartments and is widely used as a quantitative reporter of phagocytic internalization (20). Confocal imaging showed a significant increase of oAβ-engulfment in CAR-Raw264.7 (**Figure 1C**). Flow cytometry analysis also showed that CAR-Raw264.7 exhibited significant oAβ-engulfment efficiency through the quantification of oAβ-pHrodo positive cells percentage and mean fluorescence intensity (**Figure 1D**). Among these, AXL-CAR had the strongest ability of oAβ engulfment. We also introduced the human CAR constructs into the human microglial cell line HMC3, observing similarly enhanced oAβ phagocytosis in all CAR-HMC3 lines (**Figure 1E**). And AXL-CAR-HMC3 cells also had the best performance on engulfment of oAβ. These results demonstrate that TAM-based CARs can significantly boost microglial/macrophage Aβ phagocytosis *in vitro*.

### Optimizing CAR design: second-version TAM-CAR with enhanced signal transduction

**Figure 2:**
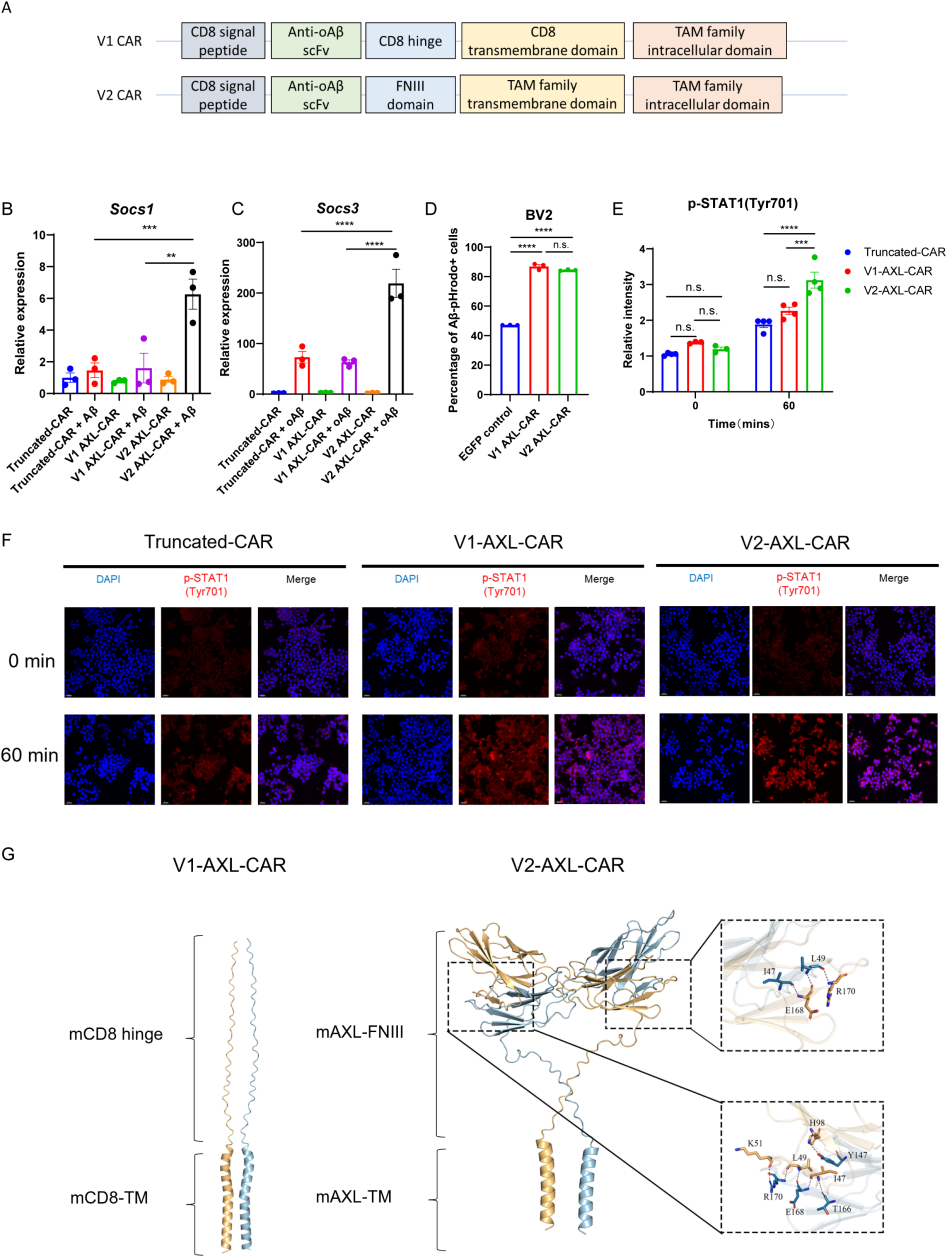
V2 CAR structure with original FNIII and TM domain transduced better signaling than V1 CAR with traditional CD8 hinge and TM domain. **(A)** Schematic representation of V1 and V2 CAR constructs. The V1 CAR design is described in **Figure 1A**. The V2 CAR consists of an Aducanumab-derived scFv, an FNIII domain, a transmembrane domain, and an intracellular signaling domain derived from a member of the TAM receptor family. **(B, C)** Relative mRNA expression levels of Socs1 **(B)** and Socs3 **(C)** in Truncated, V1-AXL-CAR and V2-AXL-CAR Raw264.7 cells after 16 h stimulation with oAβ (n=3 for all conditions). **(D)** Flow cytometry analysis of oAβ-pHrodo engulfment in EGFP control, V1-AXL-CAR and V2-AXL-CAR BV2 following 16 h incubation with oAβ-pHrodo (n=3 for all conditions). **(E)** Quantification of relative immunofluorescence intensity of phosphorylated STAT1 in Truncated and V2-AXL-CAR Raw264.7 cells following oAβ stimulation for 0 and 60 min (n=4, 3, 3, 4, 4, 4). **(F)** Representative confocal images showing STAT1 phosphorylation in Truncated and V2-AXL-CAR Raw264.7 cells at 0 and 60 min after oAβ treatment. **(G)** Structural simulation of the TM to the scFv junction in V1-CAR (left) and V2-CAR (right) with AlphaFold2. Residues involved in dimerization are labeled and shown as stick model. Cartoon models are displayed with 80% transparency. Hydrogen bonds are indicated by black dashed lines. All data are presented as mean ± s.e.m, and significance was calculated by ordinary one-way ANOVA followed by Tukey’s multiple comparisons test **(B–D)** or ordinary two-way ANOVA followed by Sidak’s multiple comparisons test **(E)** (n.s., not significant; *P < 0.05; **P < 0.01; ***P < 0.001; ****P < 0.0001).

Next, we investigated the downstream signaling induced by the CARs. We suspected that the artificial CD8 hinge-TM module may not support optimal TAM receptor oligomerization or signaling, and therefore devised a second-version (V2) CAR by replacing the native Ig-like domains of a TAM receptor with the anti-Aβ scFv, and retaining the receptor’s original FNIII repeats and transmembrane segment this time (**Figure 2A**). Importantly, qPCR analysis revealed that V2 AXL-CAR–expressing Raw264.7 cells exhibited robust upregulation of SOCS1 and SOCS3, whereas no such comparable induction was observed in cells expressing the first-version (V1) AXL-CAR (**Figures 2B, C**). To further assess whether modification of the hinge and transmembrane domains affected phagocytic function, we introduced both V1 and V2 AXL-CAR constructs into BV2 murine microglial cells. Notably, V2 AXL-CAR-BV2 cells engulfed oligomeric Aβ at levels comparable to those observed in V1 cells, indicating that alteration of the hinge and transmembrane regions had minimal impact on Aβ engulfment capacity (**Figure 2D**).

To further dissect AXL signal transduction, we directly examined downstream signaling events in truncated-CAR, V1 and V2 AXL-CAR-expressing Raw264.7 cells following stimulation with oligomeric Aβ for 60 min. Phosphorylation of STAT1 was then assessed as a readout of AXL-dependent signaling. Notably, robust STAT1 phosphorylation was detected in V2 AXL-CAR cells after 60 min of oAβ stimulation, whereas truncated-CAR and V1 AXL-CAR Raw264.7 cells exhibited less phosphorylation STAT1 activation (**Figures 2E, F**). To investigate the potential structural basis underlying the difference in phagocytic efficiency between the two constructs, we used AlphaFold2 to predict the dimeric conformations of the CARs, spanning from the transmembrane domain to the scFv junction. The predicted V2 dimer structure revealed multiple hydrogen bonds at the interface, whereas the V1 dimer lacked comparable interfacial interactions (**Figure 2G**). These results suggest that the enhanced interfacial stability of the V2 design may underlie its stronger downstream signaling and induction of anti-inflammatory regulators.

### TAM-based CAR-microglia resist inflammatory activation

**Figure 3:**
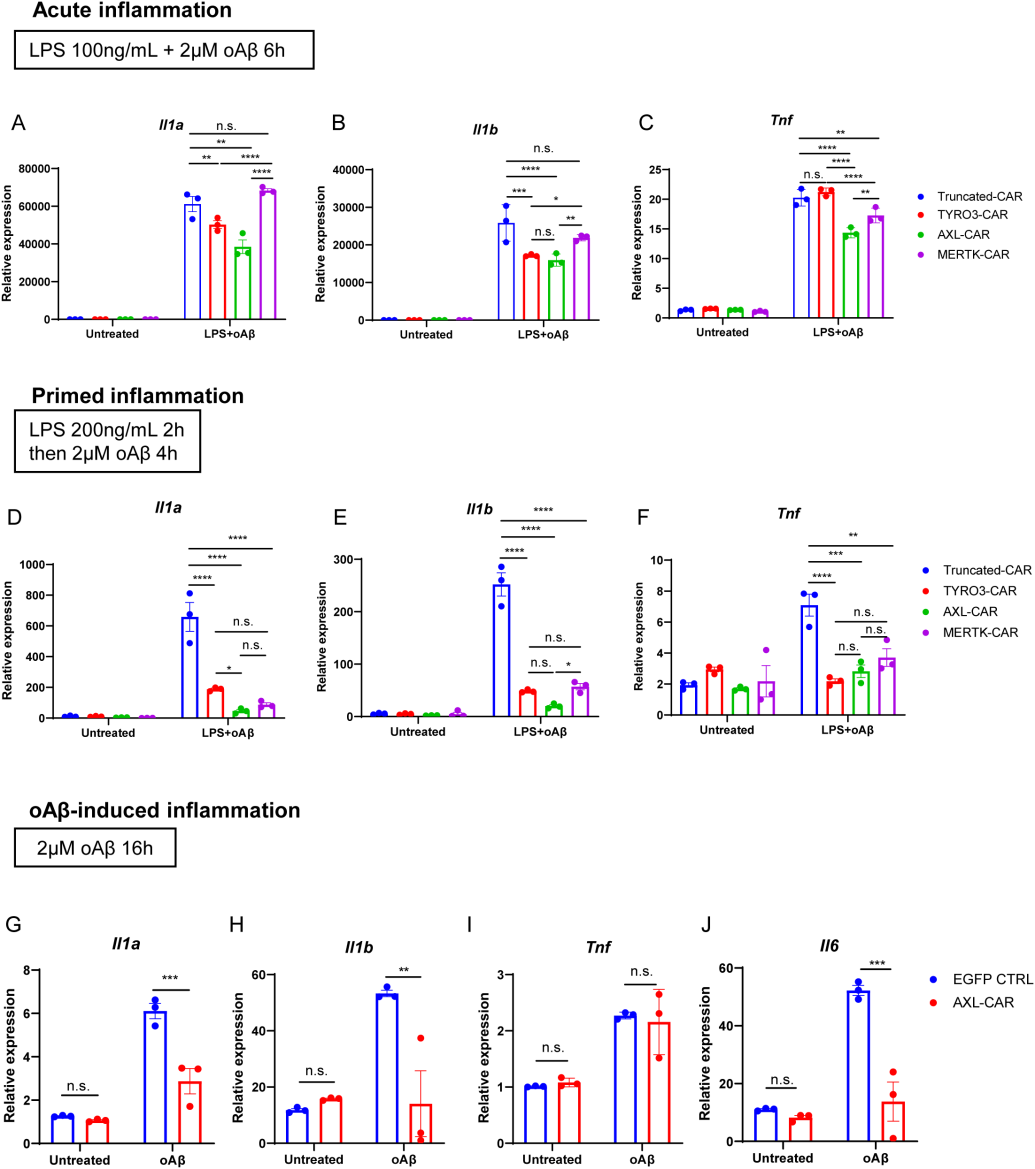
TAM receptor–based CAR-engineered macrophage or microglia exhibited enhanced anti-inflammatory activity. **(A–C)** Relative mRNA levels of *Il1a* **(A)**, *Il1b* **(B)** and *Tnf* **(C)** in Truncated, Tyro3-CAR, AXL-CAR and Mertk-CAR Raw264.7 cells after treatment with LPS (100ng/mL) and oAβ(2μM) for 6h (n=3 for all conditions). **(D–F)** Relative mRNA levels of *Il1a* **(D)**, *Il1b* **(E)** and *Tnf* **(F)** in Truncated, Tyro3-CAR, AXL-CAR and Mertk-CAR Raw264.7 cells after treatment with LPS (200ng/mL) 2h then oAβ(2μM) for 4h (n=3 for all conditions). **(G–J)** Relative mRNA levels of *Il1a* **(G)**, *Il1b* **(H)**, *Tnf* **(I)** and *Il6* **(J)** in EGFP CTRL and AXL-CAR BV2 cells after treatment with oAβ(2μM) for 16h (n=3, 3). All data are presented as mean ± s.e.m, and significance was calculated by ordinary two-way ANOVA followed by Tukey’s multiple comparisons test **(A–F)** or Sidak’s multiple comparisons test **(G–J)** (n.s., not significant; *P < 0.05; **P < 0.01; ***P < 0.001; ****P < 0.0001).

As V2 CAR design showed the strongest downstream signaling transduction based on STAT1 phosphorylation, we next tested whether TAM-based CAR-microglia are more resistant to inflammatory activation. CAR-expressing RAW264.7 cells were stimulated under three paradigms: acute co-stimulation (LPS + oAβ), LPS priming followed by oAβ, and prolonged oAβ exposure, and pro-inflammatory cytokine transcripts were measured. In the acute model, 6 h treatment of LPS+oAβ induced inflammatory gene expression in all cells (**Figures 3A–C**). Crucially, AXL-CAR Raw264.7 cells exhibited significantly reduced induction of *Il1a*, *Il1b*, and *Tnf* compared with truncated CAR controls, whereas TYRO3-CAR Raw264.7 cells showed significant reductions only in *Il1a* and *Il1b*, while MERTK-CAR Raw264.7 cells showed a significant reduction only in *Tnf* (**Figures 3A–C**). In a priming model, RAW264.7 cells were first treated with LPS for 2 h, then with oAβ for 4 h. Truncated control cells showed an increase of cytokine expression, whereas CAR-Raw264.7 cells significantly attenuated this response (**Figures 3D, E**), suggesting that CAR-engineered cells limited the amplification of inflammation following an initial stimulation. Notably, among all groups, AXL-CAR cells displayed the strongest suppression of *Il1a* and *Il1b*, together with the second strongest suppression of *Tnf* (**Figures 3D, E**). Taken together, the integrated analysis across both acute and priming conditions identified AXL-CAR as conferring the most robust anti-inflammatory phenotype. We therefore proceeded to further validate the anti-inflammatory activity of AXL-CAR in the BV2 microglial cell line. To mimic oAβ-induced inflammation, we treated with BV2 cells with oAβ alone for 16h. AXL-CAR-BV2 cells displayed a markedly blunted profile of inflammatory gene expression: *Il1a*, *Il1b* and *Il6* remained low relative to EGFP control BV2 treated with oAβ (**Figures 3G–J**). Overall, the TAM-CAR design conferred a robust anti-inflammatory response to the cells in the above situations, consistent with the known immunosuppressive roles of TAM signaling.

### Human iPSC-derived CAR-microglia-like cells showed increased Aβ uptake and anti-inflammation *in vitro*

**Figure 4:**
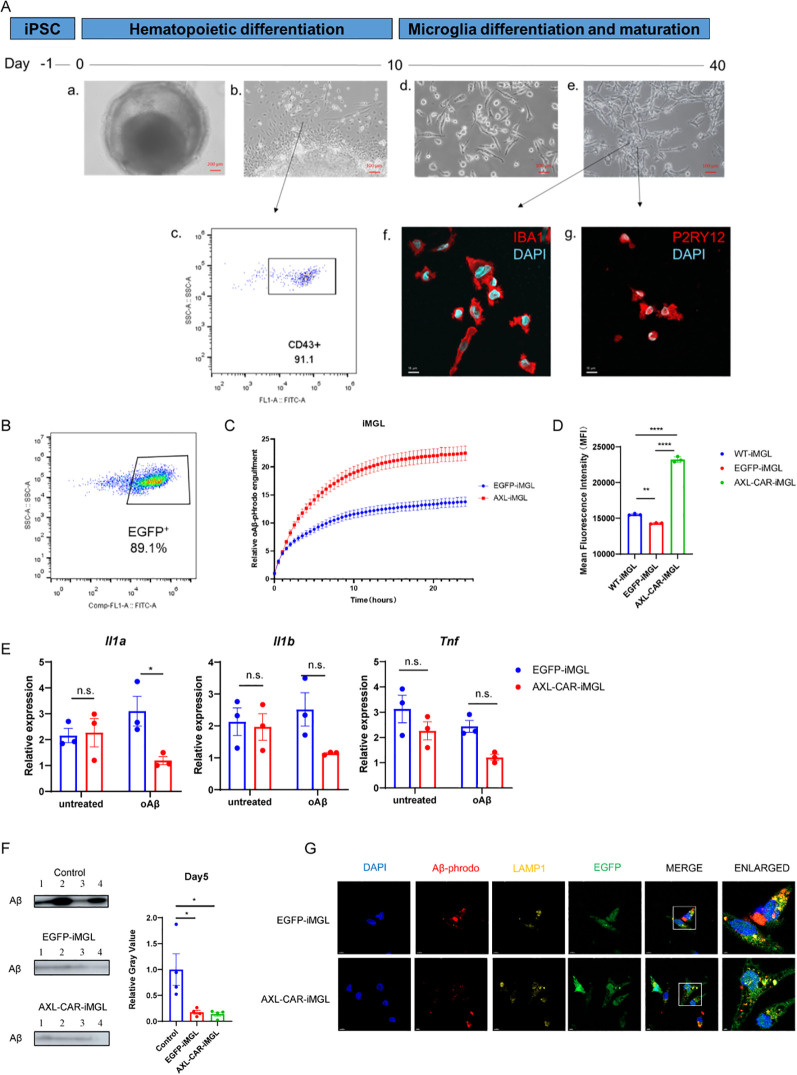
Generation of human iPSC-derived CAR-microglia-like cells (CAR-iMGLs). **(A)** Schematic overview of the differentiation process of human iPSCs into microglia-like cells (iMGLs). iPSCs were first aggregated to form embryoid bodies **(a)** and induced toward mesodermal differentiation to generate hematopoietic progenitor cells **(b)**. CD43 expression in hematopoietic progenitors was assessed by flow cytometry **(c)**. Floating hematopoietic progenitors were subsequently collected and directed toward myeloid lineage differentiation **(d)**, followed by maturation into microglia-like cells **(e)**. Expression of microglial markers IBA1 **(f)** and P2RY12 **(g)** was confirmed by confocal microscopy. Scale bars, 15 μm. **(B)** Flow cytometric analysis of CAR expression in iMGLs based on EGFP reporter fluorescence. **(C)** Live-cell time-lapse imaging of pHrodo-labeled oAβ engulfment by EGFP-iMGLs and AXL-CAR-iMGLs over 24 h in culture. **(D)** Flow cytometric analysis of oAβ-pHrodo engulfment in WT-iMGLs, EGFP-iMGLs and AXL-CAR-iMGLs after incubation with oAβ-pHrodo for 8 h (n=3 for all groups). **(E)** Relative mRNA levels of *Il1a*, *Il1b* and *Tnf* of EGFP-iMGLs and AXL-CAR iMGLs after treatment with oAβ(2μM) for 16h (n=3, 3). **(F)** Western blot analysis of residual Aβ in the culture medium following 5 days of incubation with EGFP-iMGLs, AXL-CAR-iMGLs, or in the absence of cells (control). Relative band intensities were quantified by densitometric analysis. **(G)** Representative confocal images of *in vitro*–cultured iMGLs showing Aβ-pHrodo (red), LAMP1 (yellow), EGFP (green), and DAPI (blue). All data are presented as mean ± s.e.m. For **(E)**, significance was calculated by ordinary two-way ANOVA followed by Sidak’s multiple comparisons test (n.s., not significant; *P < 0.05). For **(F)** significance was calculated by ordinary one-way ANOVA followed by Tukey’s multiple comparisons test multiple comparisons test (n.s., not significant; *P < 0.05).

To translate our approach toward human therapeutic tools, we generated CAR-microglia-like cells from human induced pluripotent stem cells (iPSCs). We transduced the AXL-CAR construct according to our selection results above, and the EGFP control constructs with lentiviral vectors into human iPSC, and differentiated the iPSCs following the process of mesoderm induction, hematopoietic progenitor specification, and myeloid differentiation protocols, based on our previously published platform (22). The collected myeloid precursor cells showed stable CD41 expression and underwent further maturation into microglia-like cells under the stimulation with IL-34, TGF-β and M-CSF (23). The iPSC-derived microglia-like cells (iMGLs) expressed primary microglia markers IBA1 and P2RY12 through immunofluorescence detection (**Figure 4A**), and showed stable CAR expression (**Figure 4B**).

To assess Aβ uptake, AXL-CAR-iMGLs and EGFP-iMGLs were incubated with pHrodo-labeled oAβ and subjected to live-cell time-lapse imaging. At 2.5 h, AXL-CAR-iMGLs began to show significant increase of oAβ engulfment compared to EGFP-iMGLs (**Figure 4C**). After 24 h culturing, AXL-CAR-iMGLs exhibited nearly two-fold higher Aβ uptake compared to EGFP-iMGLs (**Figure 4C**). Flow cytometric analysis at 8 h further demonstrated that AXL-CAR-iMGLs exhibited significantly enhanced oAβ engulfment compared with EGFP-iMGLs and WT-iMGLs (**Figure 4D**). In addition, AXL-CAR-iMGLs displayed significantly lower *Il1a* mRNA expression after 24 h of oAβ co-culture compared to EGFP-iMGLs treated with oAβ, as assessed by qPCR (**Figure 4E**). To assess whether oAβ can be degraded by iMGLs, we then incubated iMGLs with oAβ for 5 days and measured remaining Aβ by western blot. Both EGFP-iMGLs and AXL-CAR-iMGLs cultures showed marked decrease of Aβ in the coculture system, indicating effective degradation (**Figure 4F**). Immunofluorescence analysis also revealed that internalized pHrodo-oAβ colocalized with the lysosomal marker LAMP1 in iMGLs after oAβ-pHrodo treatment for 12 h (**Figure 4G**). These results demonstrate that AXL-CAR-iMGLs can effectively engulf Aβ and mediate its lysosomal degradation and without inducing an enhanced inflammatory response *in vitro*.

### Human CAR-iMGLs showed improved Aβ clearance *in vivo*

Next, we moved on to assess CAR-iMGLs *in vivo*. CSF1 is an essential cytokine for the survival of myeloid cells, however, mouse CSF1 cannot be recognized by human CSF1R (25). To improve the survival of human iMGLs in mice, we transduced the human CSF1 (hCSF1) into human iPSC and differentiate into hCSF1-iMGLs. These cells were then cultured in the medium without addition of hCSF1 for 20 days, and hCSF1-iMGLs cells showed improved survival compared with WT-iMGLs (**Supplementary Figure 1A**).

Previous studies have proved that depleting native microglia by a M-CSF1 receptor inhibitor such as PLX5622 can improve the engraftment of exogenous microglia (24, 26). Here we fed the FAD4T mouse, an established mouse model of Alzheimer’s disease (27), with PLX5622-formulated AIN-76A diet for 2 weeks. Immunofluorescence staining for IBA1 confirmed obvious microglial depletion in the PLX5622-treated group (**Supplementary Figure 1B**).

To assess the long-term Aβ clearance ability of CAR-iMGLs *in vivo*, tacrolimus was used as an immunosuppressant to mitigate xenogeneic rejection, as it is widely used in allotransplantation (28, 29). C57BL/6J mice were treated with PLX5622 for 2 weeks to deplete microglia, followed by daily tacrolimus (1 mg/kg) starting one day prior to transplantation. These mice were then intracranially injected into the hippocampus with hCSF1-expressing EGFP-iMGLs or AXL-CAR-iMGLs. (**Supplementary Figure 1C**). At 7 and 14 days post-injection, hippocampal tissues were collected and transplantation efficiency was evaluated by flow cytometry. iMGLs comprised approximately 10–15% of the total microglial population in both the EGFP-iMGL and AXL-CAR-iMGL groups at both time points (**Supplementary Figure 1D**).

**Figure 5:**
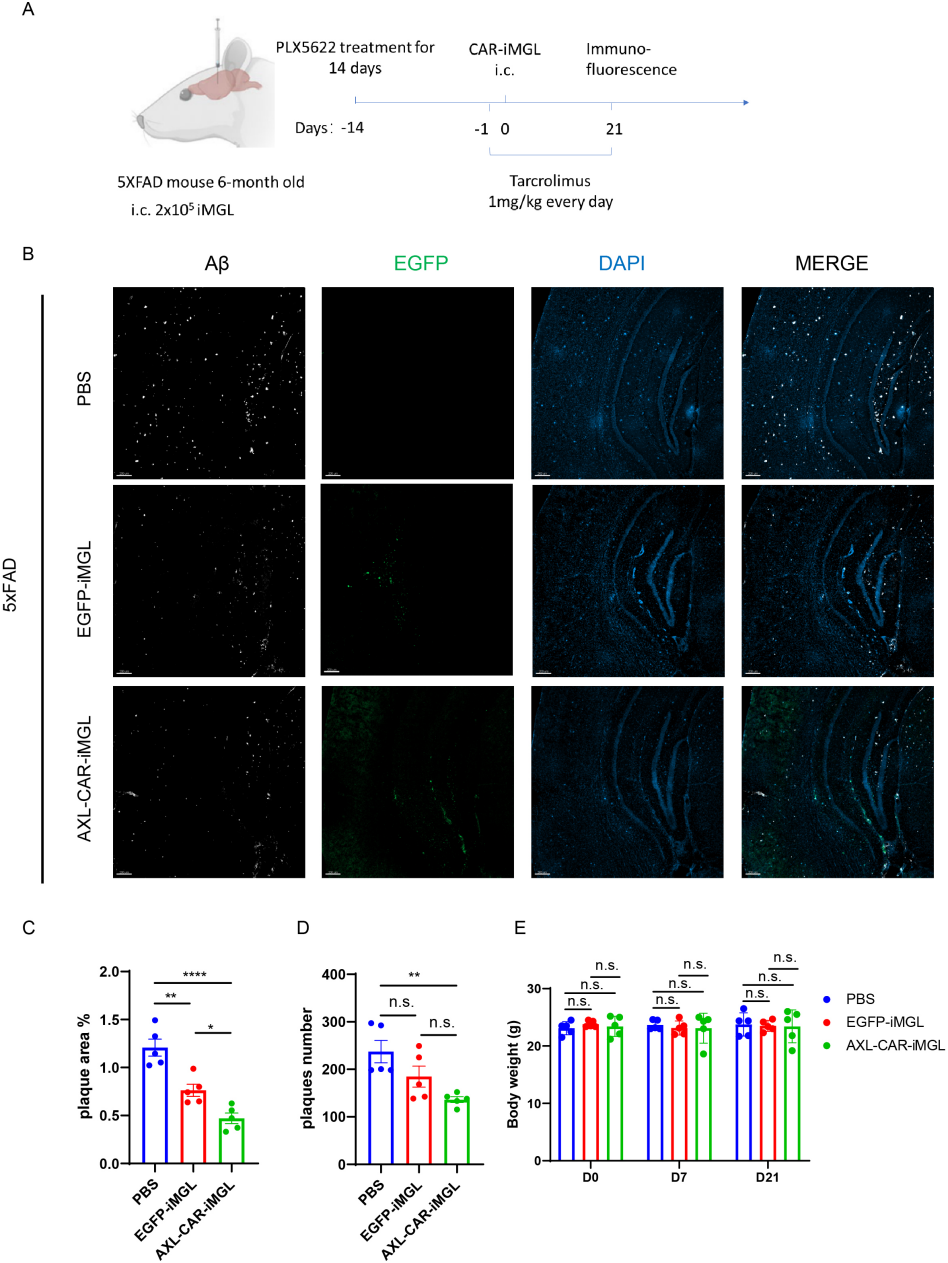
AXL-CAR-iMGLs can reduce amyloid-β burden in 5xFAD mice. **(A)** Schematic overview of the CAR-iMGLs treatment in 5xFAD mice. Briefly, 5xFAD mice were fed a PLX5622-formulated diet for 14 days, then were intracranial injected with PBS, EGFP-iMGLs or AXL-CAR-iMGLs and treated with tacrolimus (1mg/kg) daily for 21 days. **(B)** Representative confocal images of brain sections from 5xFAD mice treated with PBS, EGFP-iMGLs, or AXL-CAR-iMGLs, stained for Aβ (6E10, white), EGFP (green), and DAPI (blue). Scale bars, 200 μm. **(C, D)** Quantification of Aβ plague area **(C)** and plaque number **(D)** in brain sections from 5xFAD mice treated with PBS, EGFP-iMGLs or AXL-CAR-iMGLs (n=5 for all conditions). **(E)** Body weight of 5xFAD mice treated with PBS, EGFP-iMGLs, or AXL-CAR-iMGLs measured at days 0, 7, and 21 (n=5 for all conditions). All data are presented as mean ± s.e.m,. For **(C, D)** significance was calculated by ordinary one-way ANOVA followed by Tukey’s multiple comparisons test multiple comparisons test. For **(E)** significance was calculated by ordinary two-way ANOVA followed by Tukey’s multiple comparisons test multiple comparisons test. (n.s. not significant, *P<0.05, **P < 0.01, ****P < 0.0001).

Next, six-month-old 5xFAD mice were treated with PLX5622 for 2 weeks to deplete microglia, followed by daily tacrolimus (1 mg/kg) starting one day prior to transplantation. The mice were then intracranially injected with PBS, EGFP-iMGLs, or AXL-CAR-iMGLs (**Figure 5A**). Three weeks later, transplanted iMGLs remained detectable in the hippocampus of the treated mice, and Aβ plaques were markedly reduced in both the EGFP-iMGL and AXL-CAR-iMGL groups compared to PBS-treated controls (**Figure 5B**). Quantification of amyloid-β (Aβ) burden within the hippocampal region of coronal brain sections spanning the injection site showed that mice treated with AXL-CAR-iMGLs exhibited a significant reduction in plaque area compared with both PBS and EGFP-iMGL groups (**Figure 5C**), as well as a significant decrease in plaque number relative to PBS controls (**Figure 5D**). Consistent with the localized engraftment pattern of transplanted iMGLs, no obvious changes in Aβ plaque burden were observed in brain regions distant from the injection site. In some animals, donor-derived cells were occasionally detected along the cortical injection tract, where a localized reduction in Aβ plaques was qualitatively observed. Because these observations were sporadic, they were not subjected to systematic quantification. Importantly, mice treated with EGFP-iMGLs and AXL-CAR- iMGLs didn’t show significant weight loss in 21 days (**Figure 5E**), suggesting that CAR-iMGLs therapy has a great safety profile.

Immunofluorescence staining was performed to assess microgliosis. Compared with untreated WT mice and PBS-treated 5xFAD mice, both the EGFP-iMGLs and AXL-CAR-iMGLs-treated 5xFAD mice exhibited an increased microglial area in the hippocampus (**Supplementary Figure 2A**). The transplanted microglia displayed enlarged cell bodies and an amoeboid morphology in both EGFP-iMGL and AXL-CAR-iMGL groups (**Supplementary Figure 2B**, green arrows). In contrast, endogenous microglia showed no obvious morphological differences among EGFP-iMGLs, AXL-CAR-iMGLs or PBS-treated 5xFAD mice (**Supplementary Figure 2B**, red arrows). Three-dimensional (3D) reconstruction further revealed that endogenous microglia in WT mice exhibited a highly ramified, resting phenotype, whereas those in the 5xFAD mice showed an activated morphology characterized by shortened and thickened processes. Notably, endogenous microglia in EGFP-iMGLs or AXL-CAR-iMGLs-treated 5xFAD mice were comparable to the PBS-treated 5xFAD mice, with no additional morphological changes observed (**Supplementary Figure 2B**).

Confocal images were then used to examine the spatial relationship between microglia and Aβ and to assess cellular uptake at high resolution (**Supplementary Figure 3**). Three-dimensional reconstructions revealed that microglia exhibited complex process extension toward Aβ-positive regions, frequently surrounding or partially enclosing Aβ aggregates. The EGFP-positive transplanted microglia displayed an amoeboid morphology, while EGFP-negative endogenous microglia exhibited a more ramified morphology. Within individual IBA1-positive cells, Aβ signal was observed not only at the cell periphery but also within cytoplasmic compartments, where it overlapped with the microglial signal (**Supplementary Figure 3**). The transplanted microglia displayed an amoeboid morphology, likely reflecting the fact that iPSC-derived microglia are highly sensitive to *in vitro* culture conditions and typically exhibit a relatively activated phenotype prior to transplantation. Following engraftment, iMGLs require time to adapt to the CNS microenvironment and progressively acquire a more homeostatic microglial identity. Consistent with this finding, Xu and colleagues reported that transplanted iMGLs exhibited a primed phenotype at 3 weeks post-transplantation, characterized by relatively short, thick processes and limited branching (30). In contrast, in their study, cells analyzed at later time points (8 weeks and 6 months) gradually developed the highly ramified morphology characteristic of homeostatic microglia. Because xenogeneic grafts are prone to progressive immune rejection and eventual clearance by the host, our *in vivo* analyses were limited to the 3-week post-transplantation time point, which also restricted our ability to further investigate the interactions between transplanted microglia and the host environment.

For neuronal and synaptic integrity, NeuN and Synaptophysin (SYP) expression was assessed in the CA1 and dentate gyrus (DG) regions of the hippocampus in untreated WT mice and 5xFAD mice treated with PBS, EGFP-iMGLs or AXL-CAR-iMGLs. Although some unavoidable NeuN disruption was observed at the injection site in both PBS- and cell-treated groups, AXL-CAR-iMGLs-treated 5xFAD mice exhibited a slight but significant increased thickness of the NeuN^+^ neuronal layer in the CA1 region compared to the PBS group (**Supplementary Figures 4A**, **S4C**). In contrast, no significant differences in SYP density were observed in either the CA1 or DG regions (**Supplementary Figures 4B**, **S4D**). These results suggest that AXL-CAR-iMGLs treatment does not induce detectable neuronal or synaptic damage compared with PBS treatment.

Overall, these findings demonstrate that hCSF1-expressing AXL-CAR-iMGLs can achieve successful engraftment in the AD brain, mediate sustained and enhanced Aβ clearance *in vivo*, and do so without inducing overt neurotoxicity.

## Discussion

In this study, we developed a novel anti-inflammatory, Aβ-targeted microglia-based cell therapy strategy for AD. By engineering CAR with TAM receptor intracellular domains, our engineered AXL-based CAR-microglia-like cells showed a significant increase of engulfment of oAβ, and significant reduction in inflammatory cytokine expression. Mechanistically, engagement of the CAR with Aβ induced STAT1 phosphorylation, which in turn promotes upregulation of downstream SOCS1 and SOCS3 and thus suppressed the expression of inflammatory genes. *In vivo*, transplanted AXL-CAR-iMGLs engrafted in the AD brain, interacted with and engulfed amyloid plaques, and drove robust Aβ clearance. Thus, our TAM-based CAR design achieves the previously proposed concept of enhancing microglial Aβ clearance while avoiding the induction of neuroinflammation (20).

From a translational perspective, iPSC-derived CAR-microglia-like cells (CAR-iMGLs) therapy could overcome several limitations of current antibody-based treatments. Unlike passive monoclonal antibodies, CAR-iMGLs do not depend on Fc receptor engagement and may therefore avoid antibody-dependent cellular cytotoxicity. The iPSC source also provides a method for large-scale production of CAR-iMGLs for future application.

Several limitations should be considered when interpreting the present findings. First, the *in vivo* studies were performed in a xenogeneic transplantation setting, which may not fully recapitulate the biological interactions between engineered human microglia and the human brain. Although this model enables assessment of engraftment and therapeutic activity *in vivo*, host-versus-graft interactions and transplantation-associated immune responses may influence the functional state of transplanted iMGLs and thereby confound the interpretation of certain outcomes. In addition, donor-derived cells accounted for only a minority of the local microglial population (~15%), reflecting the current technical limitations of achieving extensive microglial replacement. Together with the relatively early analysis time point required by the limited persistence of xenogeneic grafts, these factors may have reduced the ability to fully capture the long-term therapeutic potential of CAR-iMGLs. Notably, transplanted microglia had not yet completely reacquired a homeostatic state at three weeks post-transplantation (30), which may contribute to the Aβ-clearing activity observed in control iMGLs. Furthermore, because inflammatory responses in this setting may be influenced by both engraftment-associated activation and host-versus-graft interactions, the present model does not allow definitive assessment of the *in vivo* immunomodulatory properties of CAR-iMGLs, and behavioral experiments also merit further investigation.

Future studies will therefore benefit from experimental systems that support higher levels of microglial replacement, prolonged donor-cell persistence. For example, the 5xFAD-MITRG mouse model supports long-term engraftment and expansion of human microglia while avoiding the immune rejection typically associated with xenogeneic transplantation, thereby providing a more suitable platform for investigating microglia–host interactions *in vivo* (13). In parallel, the inclusion of mechanistically distinct CAR controls, such as FcγR-based CARs sharing the same Aβ-targeting domain (31), may help define the specific contribution of AXL signaling to microglial phenotype and function. In addition, alternative delivery approaches may further enhance the translational potential of this strategy. Recent studies have demonstrated the feasibility of using intravenously administered engineered monocytes capable of infiltrating the central nervous system (12), as well as nanoparticle-based platforms that exploit calvarial monocytes for brain-directed delivery (32). Evaluating CAR-engineered myeloid cells within these emerging delivery frameworks represents an important direction for future investigation.

A second limitation lies in the pathology-specific focus of the current CAR design. While Aβ accumulation is a defining feature of AD, disease progression is driven by the convergence of multiple pathological processes, including tau aggregation and chronic neuroinflammation (8). It will therefore be important to assess the impact of CAR-iMGLs therapy on non-amyloid pathologies and to explore rational combination strategies, such as pairing CAR-iMGLs with anti-tau therapies or pharmacological modulators of neuroinflammation. In addition, validation across multiple independent iPSC lines will be required in future studies. Finally, as with all cell-based interventions, challenges related to long-term engraftment, phenotypic stability, scalability, and regulatory control must be addressed prior to clinical translation.

Recent studies have also demonstrated the feasibility of engineering microglia for Aβ targeting, including the development of “immunologically silent” CAR constructs designed to enhance Aβ phagocytosis while limiting inflammatory activation (33). Notably, similar to our TAM receptor–based design, these approaches employed another efferocytosis-related receptor, TIM4, to promote Aβ clearance with reduced immune activation. In contrast to our approach, the authors developed and screened LNP-mRNA formulations capable of crossing the blood–brain barrier, enabling *in vivo* engineering of microglia via intravenous administration and achieving effective Aβ clearance. This strategy offers a less invasive and more convenient delivery route. However, it may require repeated dosing to maintain therapeutic effects (33). By comparison, our strategy relies on transplantation of engineered microglia-like cells, which is more invasive but allows for higher local enrichment and potentially longer persistence of CAR-iMGLs within the brain, thereby supporting sustained Aβ clearance. Almost at the same time, Heiss et al. systematically screened CAR variants for Aβ engulfment in iPSC-derived microglia-like cells, although inflammatory activation was not evaluated (31). Chadarevian et al. demonstrated that engineered human iPSC-derived microglia (iMG) delivering secreted neprilysin can reduce Aβ pathology and attenuate neuroinflammation (13). Beyond microglia, gene-engineered astrocytes have also shown therapeutic promise, as Chen et al. reported that *in vivo* CAR-engineered astrocytes can effectively eliminate amyloid deposits while preserving neuronal integrity (34).

In conclusion, our study establishes a novel type of “immune-engineered” cell therapies for neurodegenerative diseases. By engineering microglia with a TAM-based CAR, we successfully integrated targeted Aβ clearance with anti-inflammatory signaling. This strategy achieved robust plaque clearance in preclinical models of AD. With further development and optimization, anti-inflammatory CAR-iMGLs may represent a promising therapeutic strategy to confront the dual challenges of protein aggregation and neuroinflammation in Alzheimer’s disease and related disorders.

## Data Availability

The original contributions presented in the study are included in the article/**Supplementary Material**. Further inquiries can be directed to the corresponding authors.

## Supplementary material

Supplementary Figure 1 Characterization of xenograft of human iMGLs into mouse brain. **(A)** CCK-8 assay assessing the viability of hCSF1-expressing iMGLs during *in vitro* culture in the absence of exogenous hCSF1 supplementation (n=6, 6). **(B)** Representative confocal images of brain sections from FAD4T mice fed a control diet or a PLX5622-formulated diet for 14 days, stained for IBA1 (red) and DAPI (blue). Scale bar, 50 μm. **(C)** Schematic overview of the iMGLs engraftment. Briefly, mice were fed a PLX5622-formulated diet for 14 days to deplete endogenous microglia, followed by daily administration of tacrolimus (1 mg/kg) starting one day prior to transplantation. EGFP-iMGLs or AXL-CAR-iMGLs expressing hCSF1 were then intracranially injected into the hippocampus. Engraftment was evaluated 7 and 14 days after transplantation by flow cytometry. **(D)** Flow cytometric analysis of the percentage of engrafted iMGLs among total microglia in hippocampus at days 7 and 14 post-transplantation (n = 3 for all conditions), Data are presented as mean ± s.e.m, and significance was calculated by multiple t test**(A)** or ordinary two-way ANOVA followed by Sidak’s multiple comparisons test **(D)** (n.s., not significant; ***P < 0.001).

Supplementary Figure 2 Characterization of microgliosis after CAR-iMGLs therapy in 5xFAD mice. **(A)** Quantification of IBA1 area in brain sections from WT or 5xFAD mice treated with PBS, EGFP-iMGLs or AXL-CAR-iMGLs (n=5 for all conditions). **(B)** Representative confocal images of Aβ (6E10, white), IBA1 (orange), EGFP (green), and DAPI (blue) in brain sections from WT or 5xFAD mice treated with PBS, EGFP-iMGLs, or AXL-CAR-iMGLs. Scale bars, 20 μm in the original images and 10 μm in the enlarged images. Three-dimensional (3D) reconstruction highlights the morphology of endogenous microglia. Scale bars, 5 μm. Data are presented as mean ± s.e.m, and significance was calculated by ordinary two-way ANOVA followed by Sidak’s multiple comparisons test (n.s. not significant, *P<0.05, **P < 0.01, ***P < 0.001).

Supplementary Figure 3 Characterization of Aβ engulfment in 5xFAD mice. Representative confocal images of Aβ (6E10, white), IBA1 (orange), EGFP (green) in brain sections from 5xFAD mice treated with PBS, EGFP-iMGLs, or AXL-CAR-iMGLs. Engulfed Aβ (yellow) was quantified by colocalization analysis of white and orange signals. 3D-reconstruction highlights the Aβ engulfment by endogenous microglia or iMGLs. Scale bars, 2 μm.

Supplementary Figure 4 Characterization of neuronal and synaptic integrity after CAR-iMGLs therapy in 5xFAD mice. **(A)** Representative confocal images of NeuN (red), EGFP (green), and DAPI (blue) in brain sections from WT or 5xFAD mice treated with WT, PBS, EGFP-iMGLs, or AXL-CAR-iMGLs. Scale bars, 100 μm in the original images and 20 μm in the enlarged images. **(B)** Representative confocal images of Synaptophysin in brain sections from WT or 5xFAD mice treated with WT, PBS, EGFP-iMGLs, or AXL-CAR-iMGLs. Scale bars, 30 μm. **(C)** Quantification of the thickness of the NeuN+ neuronal layer in the CA1 region and dentate gyrus (DG) from WT or 5xFAD mice treated with PBS, EGFP-iMGLs or AXL-CAR-iMGLs(n=5 for all conditions). **(D)** Quantification of the mean intensity of synaptophysin in the CA1 region and dentate gyrus (DG) of WT or 5xFAD mice treated with PBS, EGFP-iMGLs, or AXL-CAR-iMGLs (n=5 for all conditions). Data are presented as mean ± s.e.m, and significance was calculated by ordinary two-way ANOVA followed by Sidak’s multiple comparisons test (n.s. not significant, *P<0.05, **P < 0.01, ****P < 0.0001).

Supplementary Figure 5 Western blot images. **(A)** Western blot analysis of Aβ aggregates preparation. **(B)** Unedited western blot image of residual Aβ in the culture medium following 5 days of incubation with EGFP-iMGLs, AXL-CAR-iMGLs, or in the absence of cells (control).
