# Supplementary figures and images for: Anti-inflammatory CAR-microglia targeting Aβ for Alzheimer’s disease therapy
Source: Front Immunol. 2026 Jul 14;17:1820099. doi: 10.3389/fimmu.2026.1820099 (PMC13407183; doi:10.3389/fimmu.2026.1820099)

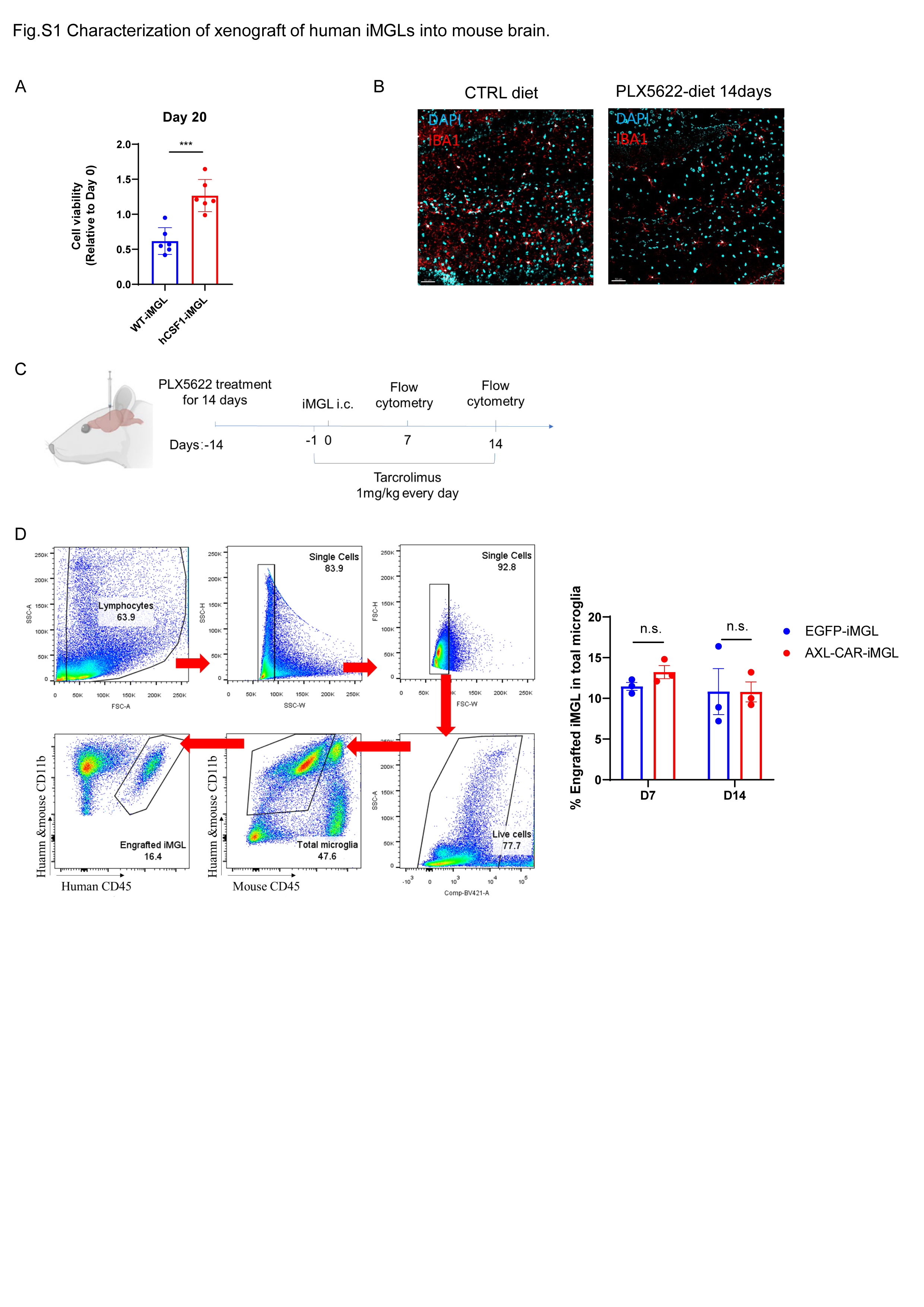

Supplement: Supplementary Figure 1 — Characterization of xenograft of human iMGLs into mouse brain. (A) CCK-8 assay assessing the viability of hCSF1-expressing iMGLs during in vitro culture in the absence of exogenous hCSF1 supplementation (n=6, 6). (B) Representative confocal images of brain sections from FAD4T mice fed a control diet or a PLX5622-formulated diet for 14 days, stained for IBA1 (red) and DAPI (blue). Scale bar, 50 μm. (C) Schematic overview of the iMGLs engraftment. Briefly, mice were fed a PLX5622-formulated diet for 14 days to deplete endogenous microglia, followed by daily administration of tacrolimus (1 mg/kg) starting one day prior to transplantation. EGFP-iMGLs or AXL-CAR-iMGLs expressing hCSF1 were then intracranially injected into the hippocampus. Engraftment was evaluated 7 and 14 days after transplantation by flow cytometry. (D) Flow cytometric analysis of the percentage of engrafted iMGLs among total microglia in hippocampus at days 7 and 14 post-transplantation (n = 3 for all conditions), Data are presented as mean ± s.e.m, and significance was calculated by multiple t test(A) or ordinary two-way ANOVA followed by Sidak’s multiple comparisons test (D) (n.s., not significant; ***P < 0.001). [file Image1.tif]

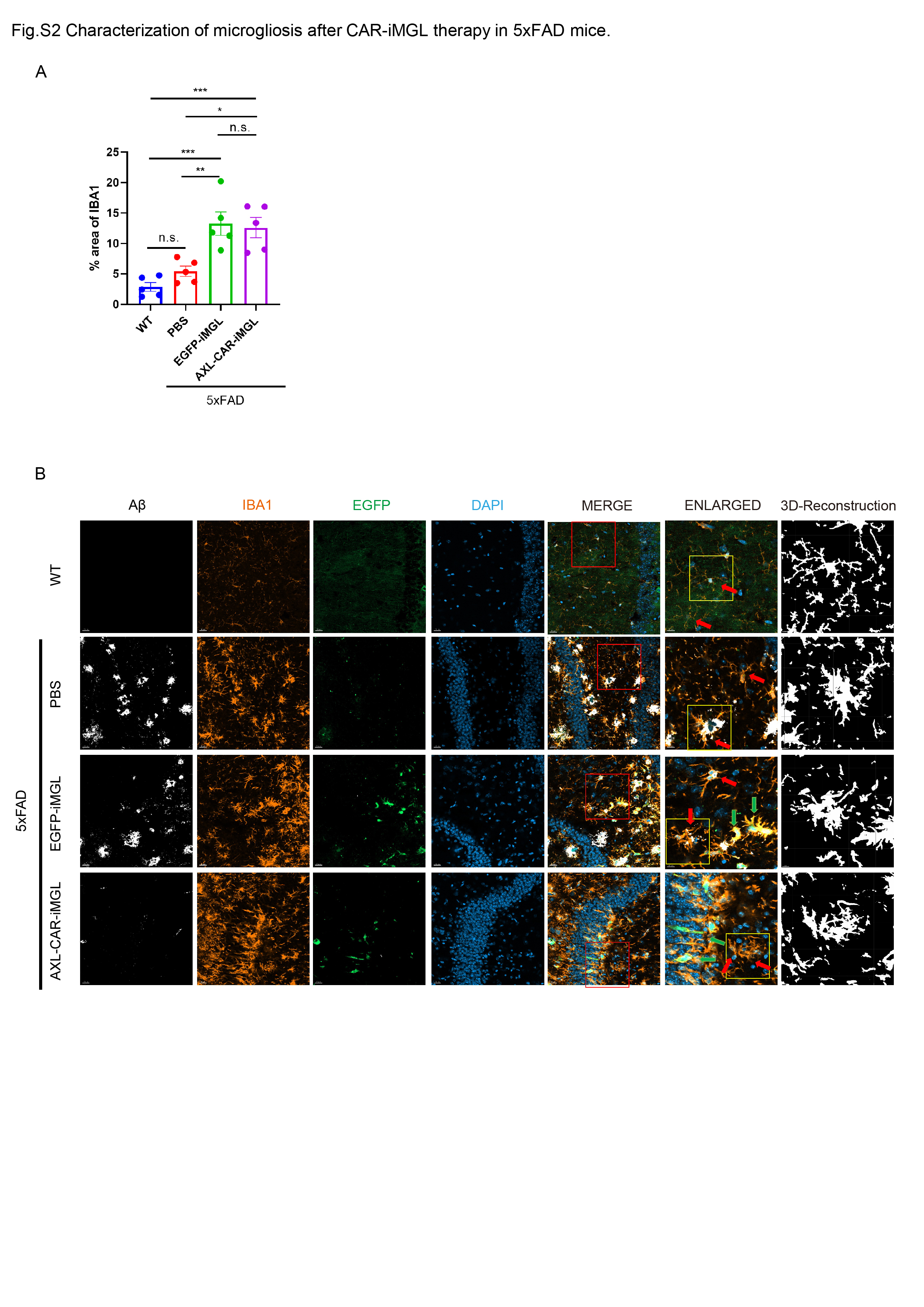

Supplement: Supplementary Figure 2 — Characterization of microgliosis after CAR-iMGLs therapy in 5xFAD mice. (A) Quantification of IBA1 area in brain sections from WT or 5xFAD mice treated with PBS, EGFP-iMGLs or AXL-CAR-iMGLs (n=5 for all conditions). (B) Representative confocal images of Aβ (6E10, white), IBA1 (orange), EGFP (green), and DAPI (blue) in brain sections from WT or 5xFAD mice treated with PBS, EGFP-iMGLs, or AXL-CAR-iMGLs. Scale bars, 20 μm in the original images and 10 μm in the enlarged images. Three-dimensional (3D) reconstruction highlights the morphology of endogenous microglia. Scale bars, 5 μm. Data are presented as mean ± s.e.m, and significance was calculated by ordinary two-way ANOVA followed by Sidak’s multiple comparisons test (n.s. not significant, *P<0.05, **P < 0.01, ***P < 0.001). [file Image2.jpeg]

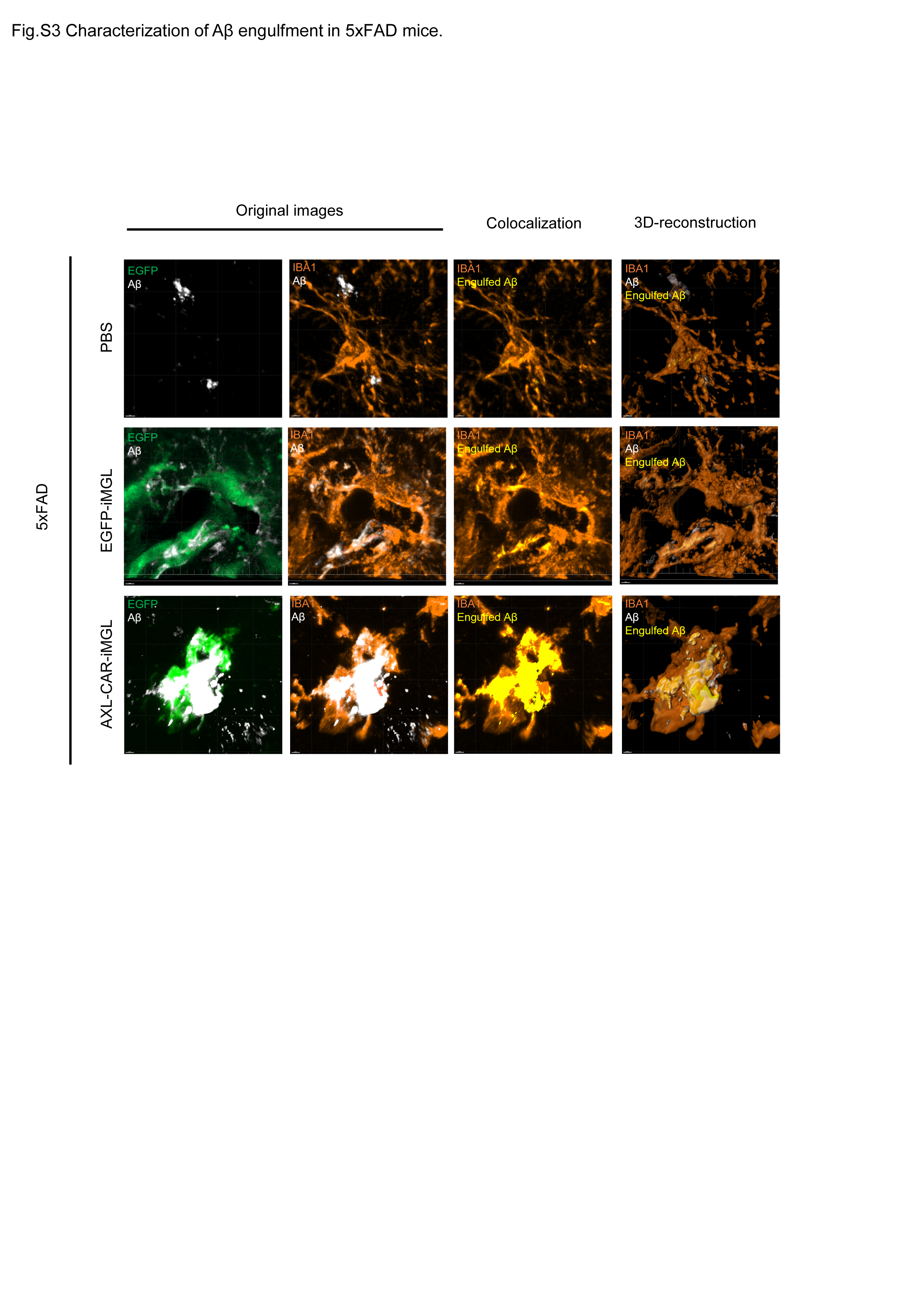

Supplement: Supplementary Figure 3 — Characterization of Aβ engulfment in 5xFAD mice. Representative confocal images of Aβ (6E10, white), IBA1 (orange), EGFP (green) in brain sections from 5xFAD mice treated with PBS, EGFP-iMGLs, or AXL-CAR-iMGLs. Engulfed Aβ (yellow) was quantified by colocalization analysis of white and orange signals. 3D-reconstruction highlights the Aβ engulfment by endogenous microglia or iMGLs. Scale bars, 2 μm. [file Image3.tif]

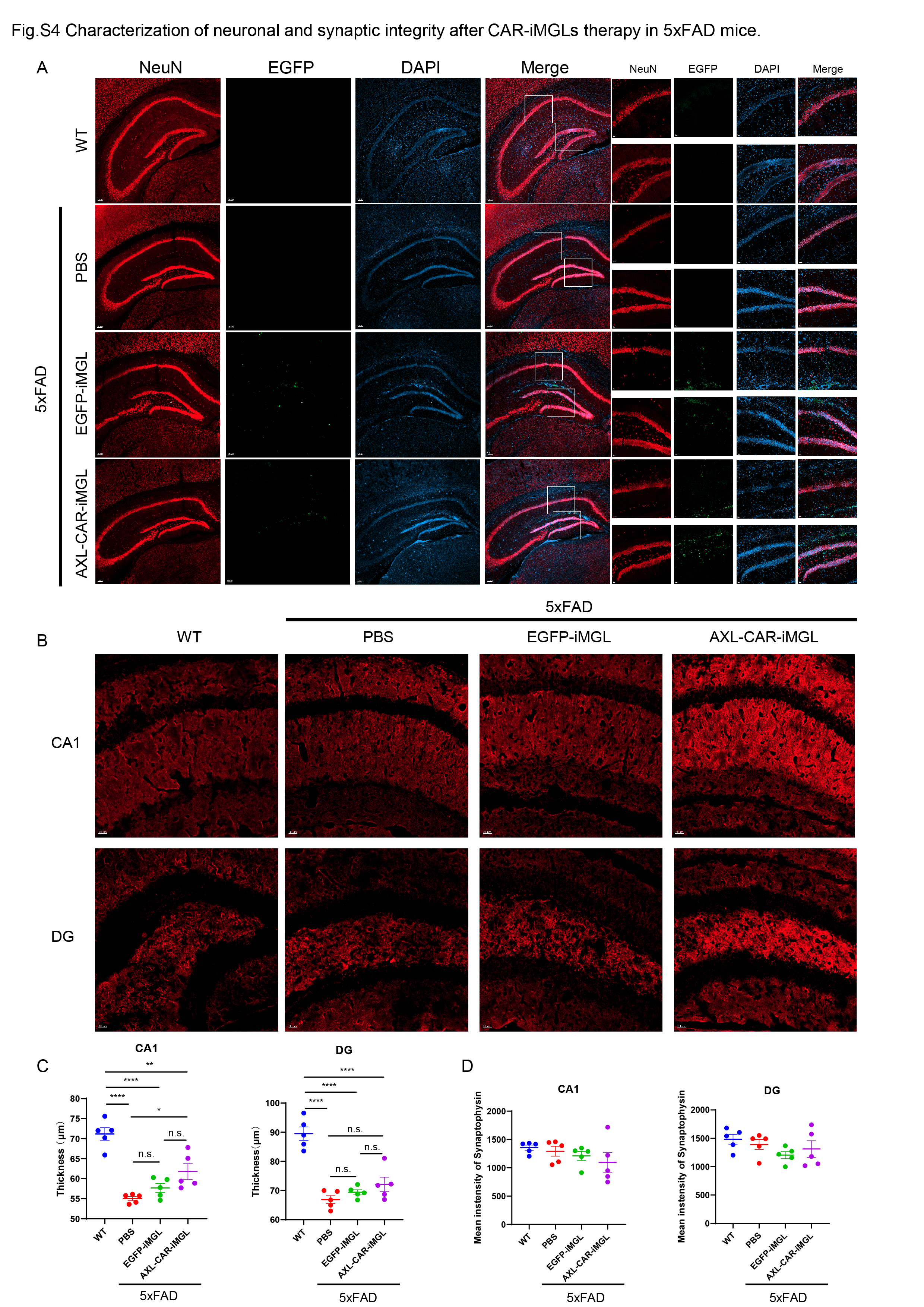

Supplement: Supplementary Figure 4 — Characterization of neuronal and synaptic integrity after CAR-iMGLs therapy in 5xFAD mice. (A) Representative confocal images of NeuN (red), EGFP (green), and DAPI (blue) in brain sections from WT or 5xFAD mice treated with WT, PBS, EGFP-iMGLs, or AXL-CAR-iMGLs. Scale bars, 100 μm in the original images and 20 μm in the enlarged images. (B) Representative confocal images of Synaptophysin in brain sections from WT or 5xFAD mice treated with WT, PBS, EGFP-iMGLs, or AXL-CAR-iMGLs. Scale bars, 30 μm. (C) Quantification of the thickness of the NeuN+ neuronal layer in the CA1 region and dentate gyrus (DG) from WT or 5xFAD mice treated with PBS, EGFP-iMGLs or AXL-CAR-iMGLs(n=5 for all conditions). (D) Quantification of the mean intensity of synaptophysin in the CA1 region and dentate gyrus (DG) of WT or 5xFAD mice treated with PBS, EGFP-iMGLs, or AXL-CAR-iMGLs (n=5 for all conditions). Data are presented as mean ± s.e.m, and significance was calculated by ordinary two-way ANOVA followed by Sidak’s multiple comparisons test (n.s. not significant, *P<0.05, **P < 0.01, ****P < 0.0001). [file Image4.jpeg]

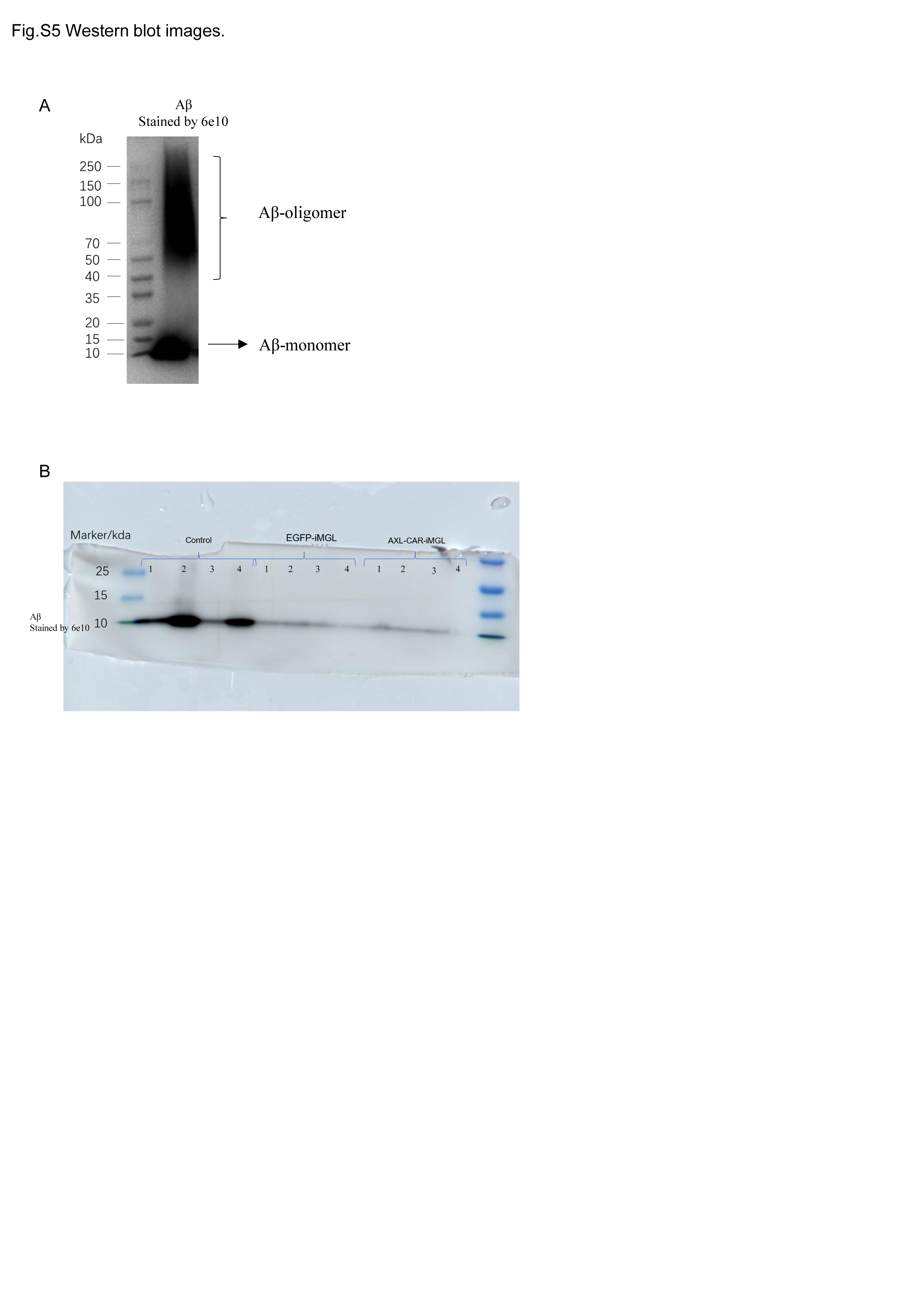

Supplement: Supplementary Figure 5 — Western blot images. (A) Western blot analysis of Aβ aggregates preparation. (B) Unedited western blot image of residual Aβ in the culture medium following 5 days of incubation with EGFP-iMGLs, AXL-CAR-iMGLs, or in the absence of cells (control). [file Image5.tif]
